# Hypersound tomography of graphitized layers buried into diamond matrix

**DOI:** 10.1016/j.pacs.2023.100528

**Published:** 2023-07-07

**Authors:** A. Yu. Klokov, N. Yu. Frolov, A.I. Sharkov, S.I. Chentsov, R.A. Khmelnitsky, V.A. Dravin

**Affiliations:** P.N. Lebedev Physical Institute of the RAS, Leninsky Prospekt 53, Moscow 119991, Russia

**Keywords:** Diamond, Picoacoustics, Hypersound, Imaging, Mechanical properties

## Abstract

Acoustic properties of buried graphitized layers in diamond formed by ion implantation followed by annealing were studied using the picosecond ultrasonic technique with spatial resolution. Two methods of elastic pulse generation were used: heating an aluminum film deposited on a diamond sample by femtosecond laser pulses and direct illumination of the graphitized layers by these pulses. We applied a multilayered model of the acousto-optical response to fit experimental results and estimate the distribution of the acoustical parameters (wave resistance, viscoelastic damping, and longitudinal sound speed) of the structures under study in depth. It was found that unique sets of spectral lines are present in the Fourier spectra of measured responses in regions with different internal structures. Mapping of the Fourier spectra made it possible to visualize regions with different internal structures. The combined use of depth profiling and mapping can serve as a tool for hypersound tomography.

## 1 Introduction

Diamond is extremely promising for photonics, optoelectronics, and MEMS devices, including those containing quantum emitters [1], [2], [3], [4], due to its properties such as rigidity, transparency in a wide range of wavelengths, and relatively low sound attenuation at frequencies of tens of gigahertz.

One of the methods for creating functional devices based on diamond is ion implantation, in which a significant concentration of radiation defects is created in the diamond matrix at a certain depth below the surface. Choosing the type of implanted ion, its energy, and radiation dose enables one to modify physical properties such as the refractive index, density, photoelastic, and mechanical properties in given thin near-surface regions in the bulk diamond [5], [6], [7], [8].

Annealing at a high temperature after implantation provides additional possibilities for structure formation. Regions with a radiation defects concentration below the graphitization threshold are restored to diamond, and above the threshold are restructured to a graphitized phase. In this case, the diamond/graphitized phase interface can be very sharp with a few nanometers root-mean-square roughness. Graphitized layers conduct electricity and effectively absorb optical radiation [9]. The elastic properties of radiation-damaged diamond, mainly its amorphous phase, were studied in various works [10], [11], [12].

The acoustic properties of graphitized layers, such as sound speed and viscoelastic damping, have not been sufficiently studied. The most suitable nondestructive method for studying acoustic properties is picosecond ultrasonics, which makes it possible to register acoustic vibrations of nanostructures with high lateral (∼1 µm) resolution. For example, in [13] this method was used to study the change in the bonding of the Au/Si interface after ion implantation; in [14], [15] the elastic interaction at the interfaces of Van der Waals heterostructures was studied; and the acoustic properties of SiGe nanolayers were studied in [16].

Tomography is used to obtain a three-dimensional distribution of a parameter of interest over a data set of smaller dimensions. Tomography methods were used in [17] to reconstruct the spatiotemporal distribution of the strain in an elastic pulse.

Hypersound tomography can be realized by methods providing information about the sample elastic properties in depth (see, for example, [18], [19]) in combination with scanning along the surface. Impressive results of depth profiling of elastic and optical properties were obtained due to time-resolved Brillouin scattering [20]. However, its successful application requires characteristic scale of the change in the elastic or optical parameters of the medium to be significantly greater than the elastic pulse spatial extension. In many cases, it is compulsory, since it is not always possible to excite an elastic pulse with a length of less than 10 nm. At the same time, it is possible to achieve nanometer accuracy in determining the layer thicknesses in layered structures with multiple reflections of elastic pulses [16], [21].

In this work, we studied the propagation of hypersound elastic waves (picosecond elastic pulses) in the structures with buried graphitized layers obtained by implanting carbon ions (C^+^) with various doses into a diamond matrix and subsequent annealing at high temperatures. We used two methods: the first was probing by elastic pulses generated by femtosecond optical excitation of an aluminum film deposited on the sample, and the second was based on the elastic pulses generation by the graphitized layers themselves upon their optical excitation. We used the maximum likelihood method to profile the acoustic properties by building the one-dimensional layered structure model and fit its response to laser excitation. The acoustic and optical parameters of the layers were chosen by the least squares method to achieve the best agreement with the experiment.

## 2 Material and methods

### 2.1 Samples

**Fig. 1:**
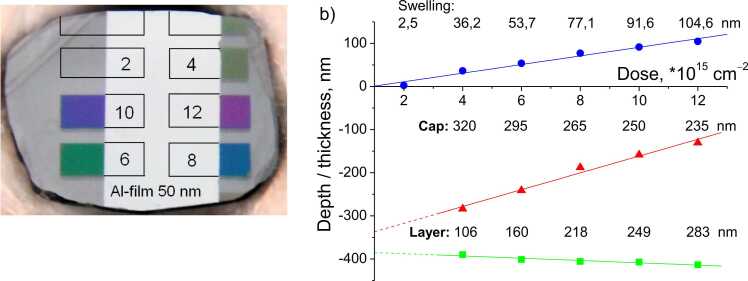
a) The photograph of the diamond sample surface with graphitized layers (colored areas) and an evaporated aluminum film (white area). The coloration of areas with different doses of implantation is caused by the light interference in a thin diamond layer covering the graphitized layer. Thin lines indicate the implantation areas boundaries under the aluminum film. The numbers indicate the implantation dose: 4, 6, 8, 10 and 12∙10^15^ cm^−2^. b) The upper (blue) line shows the amount of diamond surface swelling above the graphitized layer, the middle (red) line shows the position of the graphitized layer upper boundary, and the lower (green) line shows the lower boundary position. The numbers for each dose indicate the magnitude of swelling, the diamond cap layer thickness and the graphitized layer thickness.

For the preparation of two samples, referred to below as DR230 and DR232, polished natural ∼400 µm thick diamond plates (type Iab) with the (001) orientation were selected. The diamond surface roughness according to AFM was less than 4 nm. C^+^ ions with energy of 350 keV and doses from 2∙10^15^ cm^−2^ to 12∙10^15^ cm^−2^ were implanted into the plate through a set of masks. It is known that, in this case, a non-uniform defects distribution with a clear maximum at ∼400 nm depth is created in the near-surface region [9]. Regions with a defect concentration below the threshold value (7∙10^22^ cm^−3^ for the C^+^ ion) are restructured into diamond, and regions with that above the threshold value are restructured into a graphitized phase during subsequent high-temperature (∼1500ºС) annealing in vacuum. As a result, graphitized diamond layers with ∼100–300 nm thickness were formed at 200–400 nm depth, see Fig. 1a,b. The layers thicknesses, their depth, and the boundaries' roughness were determined using spectral ellipsometry and transmission spectra measurements similar to [22]. It appeared that the layers have sharp boundaries with ∼4 nm roughness (RMS). The complex refractive index was 2.310 + 0.878i at 800 nm and 2.263 + 0.821i at 400 nm. A ∼50 nm thick aluminum film was deposited onto a part of the sample's surface by thermal evaporation in a vacuum. Thus, it was possible to excite either the layer itself or the aluminum film above it with laser pulses to generate a hypersound pulse.

We used the data on diamond swelling in the implantation area and the "piston" model [23] to estimate the density of the graphitized layer. The obtained values of the graphitized layer's density are shown in Table 1. Thus, the density of graphitized layers decreases by approximately 5% with an implantation dose increase.

**Table 1:** Average density of the graphitized layer ρ depending on the implantation dose.

Dose, 10^15^ cm^−2^	4	6	8	10	12
ρ, 10^3^ kg/m^3^	2.32 ± 0.05	2.34 ± 0.05	2.28 ± 0.05	2.23 ± 0.05	2.22 ± 0.05

### 2.2 Experiments

**Fig. 2:**
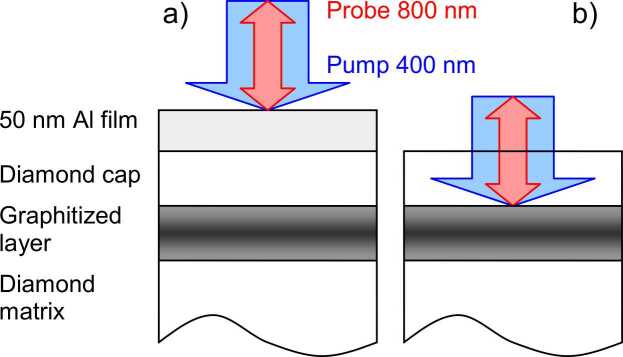
Sample structure and experimental scheme. a) Hypersound pulse generation by optical excitation of aluminum film, b) Direct excitation of hypersound by pulse illumination of buried graphitized layer.

We used a two-color pump-probe technique to study the acoustic properties of the samples (Fig. 2). The pulses of the Mira-900 femtosecond laser (λ = 800 nm, τ = 150 fs) were divided by a beam splitter in a ratio of 10:1 into two channels – excitation and probing. A BBO-crystal as a second harmonic generator (λ = 400 nm) was installed in the excitation channel. A controlled delay line and a Sagnac interferometer [24] were installed in the probing channel, allowing us to register a time-resolved response: small relative changes ΔR/R in the complex reflection coefficient R= |R|·e^iφ^ of the sample caused by the hypersound pulses propagation. An electro-optical modulator was installed in the excitation channel to realize synchronous detection. Thus, the direct results of the measurements were the time-resolved dependences: Re(ΔR/R)= Δ|R|/|R| – amplitude of the response and Im(ΔR/R)= Δφ – phase of the response. The sizes of the focal spots of the pumping and probing beams were ∼2 µm (FWHM). The samples were placed on a 3D table for response mapping with 1 µm resolution. For a more detailed optical scheme of the experimental setup see [25].

## 3 Results

### 3.1 Time-resolved responses

**Fig. 3:**
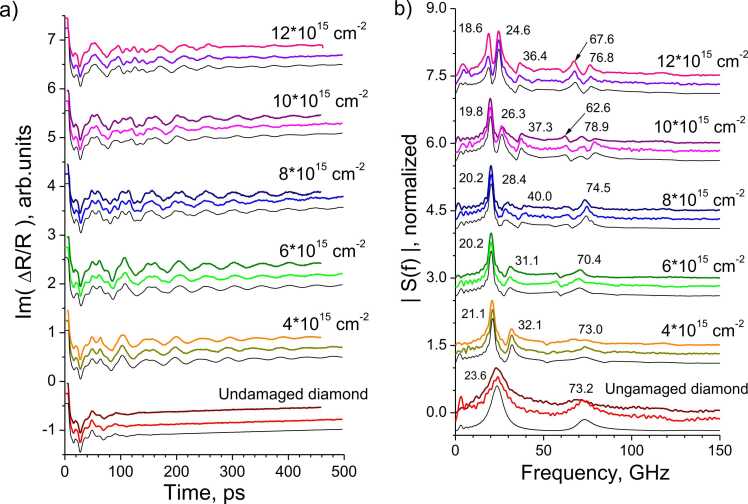
Time resolved responses (a) and spectra (b) measured at different regions. Aluminum film was used as an elastic pulse generator. a) Phase of the responses, measured on samples DR232 (upper curves) and DR230 (middle curves). b) Corresponding spectra after subtracting the slow thermal component. Thick curves (color online) are experimental. The thin black lines in both figures show the simulation results.

The first series of experiments consisted in measuring the time-resolved phase changes of the reflection coefficient on regions with different implantation doses and on undamaged diamond using an aluminum hypersound generator. The response was caused by a displacement of the aluminum film surface and a change in the complex refractive index as a result of both the arrival of elastic pulses and heating of the film. Therefore, the responses (Fig. 3a) have two components: a slowly decreasing thermal component and oscillating one. The first peak (16 ps) in all responses is caused by the rapid hot carrier’s diffusion to the aluminum-diamond interface and the elastic pulse formation there [26]. It should be noted that for the first 15 ps, all responses coincide. Further, the responses on the graphitized areas begin to differ significantly from that on the undamaged diamond. This suggests that the layers effectively reflect hypersound elastic pulses. The responses in areas with different implantation doses differ from each other, since these areas have different resonant frequencies, depending on the thicknesses and acoustic properties of their constituent layers.

Fig. 3b shows the Fourier spectra of the responses, presented on Fig. 3a after subtracting the thermal component. Two spectral lines frequencies of 23.6 and 73.2 GHz on undamaged diamond differ significantly from the estimate of the eigen frequency of the aluminum film according to the acoustic mismatch model (V_Al_/4d_Al_∼ 31 GHz). This fact suggests that the contact between the aluminum and the diamond is not rigid.

The spectra obtained on graphitized regions also contain low-frequency and high-frequency peaks, but they have a more complex structure. These peaks are due to the elastic pulse propagation over the entire structure and its reflection peculiarities at the layer boundaries.

The doublet shape of the peak in the region with 12∙10^15^ cm^−2^ implantation dose draws special attention. This shape is due to the fact that the spectral components of the pulses reflected from aluminum/diamond interface and from the diamond cap and graphitized layer boundary come approximately in antiphase to the aluminum surface at 21 GHz frequency. In general, it can be seen that the response spectra measured in areas with different implantation doses differ significantly from each other, and therefore can be used to reveal the structural features of the samples.

**Fig. 4:**
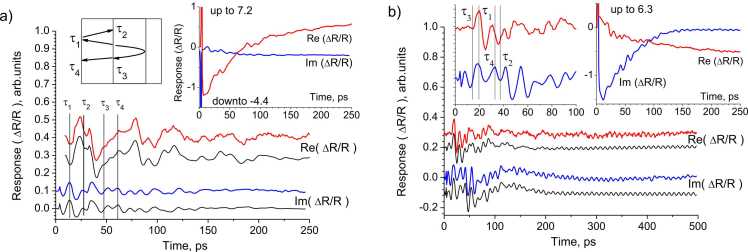
Real and imaginary components of the response oscillating part obtained by graphitized layer direct irradiation: the experiment (upper curves) and the simulation results (lower curves). The vertical lines mark the strain pulses arrival times τ_1_ - τ_4_ shown in the diagram above. On the inset: experimentally obtained responses before subtracting the thermal part. a) 12·10^15^ cm^−2^ dose, b) 4·10^15^ cm^−2^ dose.

The next series of experiments was devoted to measuring the graphitized layers responses to pulsed optical excitation. In this case, light absorption occurs directly in the graphitized layer. The generated strain pulse in the diamond cap is exponential with a spectral width of 100 GHz, while in the graphitized layer it is bipolar due to the thermoelastic effect. The right insets in Figs. 4a and 4b show the amplitude and phase of the response for regions with 4·10^15^ cm^−2^ and 12·10^15^ cm^−2^ implantation dose on the DR232 sample. The recorded responses also consist of slow and fast oscillating components. The sharp peak at the initial moment is caused by hot photo-generated carriers in the graphitized layer. The fast oscillating component is shown in the main part of Fig. 4a,b.

The complex and seemingly chaotic type of responses is due to the fact that the optical response is recorded from the entire structure, in which a set of several elastic pulses propagates. The vertical lines mark the arrival times of deformation pulses τ_1_ − τ_4_, shown in the left inset in Fig. 4a. At these moments of time, the phase of the oscillations is disrupted (Fig. 4b) or the shape of the response changes (Fig. 4a). Fig. 4b shows long oscillations with a frequency of ∼100 GHz, which correspond to Brillouin scattering on an elastic pulse propagating from the graphitized layer into the bulk diamond.

**Fig. 5:**
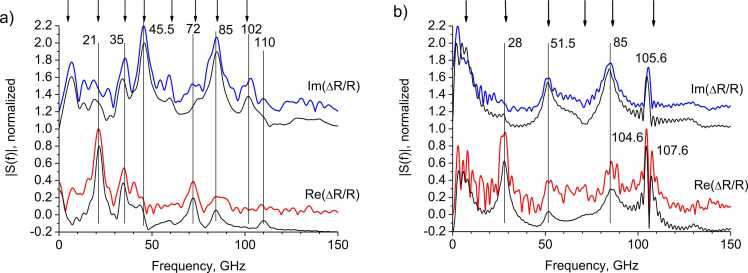
a) 12·10^15^ cm^−2^ dose, b) 4·10^15^ cm^−2^ dose. Spectra of the phase and amplitude of the responses oscillating part in the case of direct illumination of the graphitized layer: the experiment (upper curves) and simulation results (lower curves). The vertical lines and numbers mark the experimental peak frequencies. The vertical arrows indicate calculated eigen frequencies of the structure vibrations (Fig. 5a: 4.8, 21.1, 33.5, 45.8, 60.7, 73.4, 85.1, and 101.5 GHz; Fig. 5b: 7.25, 28.4, 51.7, 71.4, 86.3, and 108.4 GHz).

Fig. 5a,b shows the Fourier spectra of the responses, presented in Fig. 4a,b after subtracting the thermal component. It can be noted that the spectral lines for the amplitude and phase of the response do not completely coincide with each other. For example, for 4·10^15^ cm^−2^ dose, peaks at 51.5 GHz and 85 GHz are present both in the amplitude spectrum and in the phase spectrum, while oscillations with frequencies of 28 GHz and 107.6 GHz appear only in the amplitude spectrum. For 12·10^15^ cm^−2^ dose, peaks at 45.5 GHz and 102 GHz are appears in the phase spectrum, while peak at 21 GHz appear only in the amplitude spectrum. A similar situation is observed for other implantation doses.

The 104.6, 107.6 GHz doublet in the amplitude spectrum attracts attention. The 104.6 GHz line corresponds to Brillouin scattering in the diamond bulk, while the 107.6 GHz peak, as will be shown below, corresponds to the eigen oscillations of the structure.

### 3.2 Hypersound tomography

**Fig. 6:**
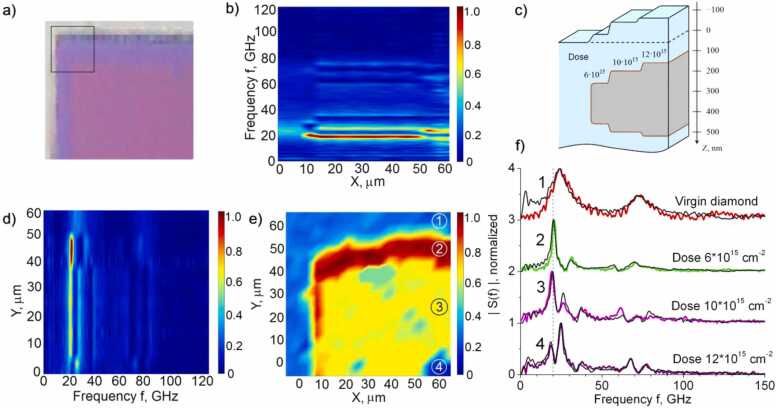
a) The optical photograph of the implantation area edge with 12∙10^15^ cm^−2^ dose. The black square shows the scan area. Different shades correspond to the heterogeneity of the implantation dose. b) Response spectrum versus position along the horizontal line with Y= 0 at e. c) The scheme of the internal structure of the sample, indicating the graphitized layers depths. d) Response spectrum versus position along the vertical line with X = 60 µm at e. e) Mapping the edge of the implantation area with 12∙10^15^ cm^−2^ dose using picosecond acoustics at 20 GHz frequency. Numbers denote points whose response spectra are shown by colored lines in f. The black lines in f show the spectra measured on undamaged diamond and regions with implantation doses of 6, 10, and 12∙10^15^ cm^−2^ dose. The dotted line shows the frequency of the mapping shown in e.

As shown, the response spectrum depends significantly on the implantation dose. This allowed us to visualize the internal structure of the sample from the spatial distribution of the selected spectral components. Fig. 6a shows an optical photograph of the implantation area edge with a dose of 12∙10^15^ cm^−2^. The dose was accumulated in a series of sequential implantation processes through the masks, which led to their slight displacement. Due to this fact, a zone with a stepwise dose was formed, as schematically shown in Fig. 6c. On an optical photograph (Fig. 6a), this is shown by a change in color. The black square marks the scanning area 60 × 60 µm^2^ in size. During scanning, the response was recorded at each point, its slow thermal component was subtracted, and the Fourier transform of the fast component was performed. Fig. 6f shows the distribution of the spectral component magnitude at 20 GHz frequency over space. It can be seen that the regions with different doses are visualized with a resolution better than 2 µm. Fig. 6b and Fig. 6d present the spectrum transformation when scanning horizontally at Y= 0 µm (Fig. 6b) and vertically at X = 60 µm (Fig. 6d). The thin lines correspond to spectral peaks. Significant changes in the spectra are clearly visible when crossing the boundaries of the areas with different implantation doses.

Fig. 6 f compares the response spectra recorded at the points marked in Fig. 6e with digits 1–4 to the responses spectra recorded earlier on undamaged diamond and areas with doses of 6, 10, and 12∙10^15^ cm^−2^. The vertical line at 20 GHz corresponds to the mapping frequency. The coincidence of the spectra enables us to determine the dose of implantation at a given point. Thus, in Fig. 6f, the complex structure at the edge of the graphitization region was revealed by picosecond acoustics.

## 4 Discussion

Profiling the acoustic and structural sample parameters in depth requires constructing a model for elastic and optical pulse propagation in an inhomogeneous medium. The corresponding theory was developed in [18]. An essential condition for this model is the elastic pulse spatial extension small compared to the acoustic and structural inhomogeneities scale. In diamond-based structures similar to the one we studied, this requires elastic pulse generation with less than 5 ps duration, which is difficult to implement. In the case of a longer pulse, the response is the result of simultaneous scattering on different sample parts and therefore becomes difficult to interpret. In this work, we modeled the structures with a set of layers, the number and parameters of which were chosen so as to fit the response best. Since the dimensions of the focal spots of the pumping and probing beams were ∼2 µm (FWHM), while the thickness of the structure was less than 0.5 µm, we assumed that the diffraction effects were insignificant and used a one-dimensional model.

The elastic pulse propagation and its interaction with light are considered in [27]. However, we used the approach in the "frequency domain", similarly to [16], to calculate the excitation and propagation of elastic pulses in a layered structure. The equations of motion were analytically solved in the frequency domain, and then a transition to the time domain was made using the discrete Fourier transform. And it was the time dependences that were compared with the experiment. Such an approach makes it easy to take into account non-rigid contact between layers [15], for example, aluminum and diamond, the presence of a concentrated (δ - distributed) mass at the boundaries of layers, for example, monolayer of materials with high density, as well as viscoelastic sound damping.

The optical response was calculated similarly to [28]. In simulation, we assumed the fact that the response from deeper regions of the structure is detected at large delay times. This allows us to consistently increase the number of variable parameters, considering the previously obtained parameters as known. Such a layer-by-layer fit enables us to increase the parameter determination reliability and reduce the simulation time.

First, when processing the experimental data presented in Fig. 2a, the response of the aluminum film to undamaged diamond was fitted. Mechanical contact with the substrate was modeled using a "weightless springs" model as in [15]. The photoelastic, photothermal, and thermal parameters were determined, as well as the rigidity of the elastic bond with the substrate and hypersound damping. The simulation showed that the aluminum film thickness of 53.0 ± 0.5 nm corresponds to the growth values, the rigidity of the elastic bond with diamond is η = 1.8·10^19^ N/m^3^ (characteristic frequency is f_0_ =η·(Z_1_ +Z_2_)/(2πZ_1_Z_2_)= 220 GHz), and viscoelastic damping length is 4.5·10^16^/ω^2^ [m]).

**Fig. 7:**
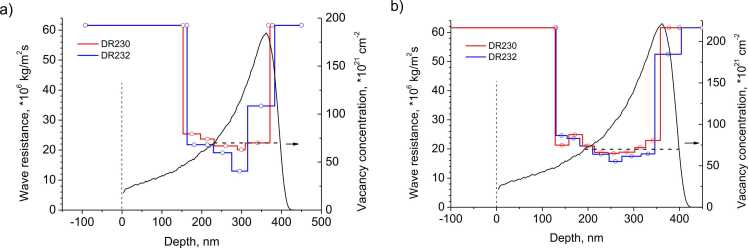
Depth profiling of the wave resistance of the graphitized layer (broken lines) for implantation doses of 10∙10^15^ cm^−2^ (a) and 12∙10^15^ cm^−2^ (b). Depth is measured from the position of the surface of an undamaged diamond (vertical dashed line). The smooth black line is the calculation of the vacancies concentration by the TRIM program for implanting a C^+^ ion with an energy of 350 keV into diamond. The horizontal dashed line shows the threshold concentration for graphitization during annealing.

Next, we introduced a layer into the model, its depth and thickness corresponded to the data in Fig. 1b. The diamond cap layer thickness, as well as the sound speed and graphitized layer thickness, varied for each implantation dose. The sound speed and cap layer density were assumed to be equal to diamond, and the graphitized layer density was taken from Table 1. It was assumed that the boundary between the layers can be described using an acoustic mismatch model (AMM). Calculations showed that the experimental results are well fitted by a single layer for implantation doses less than 10^16^ cm^−2^. To improve agreement with experiment at high implantation doses additional layers were introduced. The partitioning was as follows. Let us assume that estimates of the thickness and sound speed in the layer are obtained as the result of the fitting. This layer was divided into two equal parts, where the sound speeds were assumed to be the same. The experimental response was re-fitted, and the sublayers thicknesses and the sound speed in them could vary independently. Further, the partitioning procedure can be applied to the sublayers until the response approximation error became weakly dependent on the number of layers. We also investigated the dependence of the thickness and velocity estimates on the given layer density. It emerged that the product of velocity and density, i.e. the wave resistance, as well as the ratio of the layer thickness to the velocity, vary by no more than 3% when the density changes by ± 30%. This result is not surprising, since the acoustic properties of the layer depend on two parameters, the elastic wave propagation time through it (i.e. the ratio of thickness to sound speed) and the wave impedance [16]. Good agreement between calculation and experiment was achieved by dividing the layer with a dose of 10·10^15^ cm^−2^ into five, and with a dose of 12·10^15^ cm^−2^ into eight sublayers (∼30–40 nm average thicknesses). The introduction of a small sound damping associated with the phonon viscosity allowed us to reach an even better agreement between the calculation and experiment. According to our estimates, the viscoelastic damping length in the graphitized layer is 1.6·10^18^/ω^2^ [m], which corresponds to a 100 GHz phonon lifetime of ∼400 ps. The calculated responses and spectra are shown in Fig. 2a,b as black curves, excellent agreement with the experiment can be noted. From the known density and longitudinal velocity, the elasticity tensor component C_11_ – longitudinal elastic modulus – was calculated. The calculation results are shown in Table 2, and the wave resistance profiling in depth is shown in Fig. 7. The zero depth position corresponds to the diamond surface before implantation. The smooth line shows the calculation result of diamond radiation damage (vacancy concentration) by the Monte Carlo method in the TRIM program for C^+^ ion implantation with 350 keV energy and the corresponding doses.

**Table 2:** Characteristics of buried layers obtained from the analysis of picoacoustic measurements.

Dose cm^−2^, × 10^15^	Depth, nm		Thickness, nm		Sound velocity, μm/ps		Wave resistance Z, kg/m^2^s × 10^6^		C_11_, GPa	
Sample	230	232	230	232	230	232	230	232	230	232
4	322	320	93	83	11.3	11.6	26.2	27.0	296	312
6	297	289	134	119	10.9	11.0	25.5	25.7	278	283
8	247	286	170	167	10.4	10.3	23.7	23.5	234	241
10	245	260	209	209	10.1	9.03	22.5	20.10	228	182
12	235	235	260	270	9.32	9.76	20.7	21.7	193	211

The horizontal dashed line marks the depths at which the defect concentration exceeds the graphitization threshold and, accordingly, graphitization occurs during annealing. Since the graphitized diamond density is 1.6 times less than that of the original one, this region expands in the direction of lower resistance (i.e., from the depth to the surface) and swelling occurs. Accordingly, the position/location of the graphitized region leading edge and the position/location of the maximum radiation damage region are shifted to lower values, and the position/location of the sample surface after implantation and annealing corresponds to negative depth values.

The broken lines in Fig. 7 show the dependence of wave resistance on depth obtained for samples DR230 and DR232 with implantation doses of 10∙10^15^ cm^−2^ and 12∙10^15^ cm^−2^. It can be seen that the back side of the graphitized layer corresponds to the radiation damage calculations, and the front side is shifted towards shallower depths. The position of the maximum radiation damage area is also shifted towards shallower depths, and it is in this area that we observe the minimum wave resistance.

In order to estimate the depth resolution, we estimated the errors in the joint determination of the layer depth and the layer thickness. To do this, we calculated the error covariance matrix. It turned out that under the experimental conditions, the root-mean-square (RMS) error of the layer depth ranged from 0.6 to 1.1 nm. The maximum root-mean-square error in determining the layer thickness for the thickest layer (dose 12∙10^15^ cm^−2^) did not exceed 6 nm. For thinner layers (lower radiation doses), it was less. Such small error values are associated with multiple passages of elastic pulses through the structure. The thickness of the structure is ∼500 nm, the transit time of the elastic pulse is ∼50 ps. At the same time, the duration of the response is up to 500 ps, which means tenfold passage of an elastic pulse through the structure. Therefore, when modeling, even small deviations in the layer thicknesses lead to a significant deviation of the calculated response from the experimental one at long times.

To determine the full set of acoustic parameters (speed of sound, thickness), we needed to know the density of graphitized layers. In the most general case, when there is no priori information about the sample, within the framework of the described technique, the sample can be modeled as a set of a finite number of layers and an elastic half-space behind them. If the structure is opaque, then the acoustic parameters of the layers are wave impedance and sound propagation time. It is these parameters that can be obtained as a result of fitting. If the sample is semi-transparent, then the additional parameters will be the complex refractive index, the thickness, and the photoelastic parameters of the layers.

The simulation of optical excitation and propagation of elastic pulses in diamond with a graphitized layer was also carried out. The layer parameters corresponded to those determined above in experiments with an aluminum generator (see Table 2). To achieve the best agreement with the experiment, the longitudinal wave velocity, thickness, and depth of the layer were varied within small limits of the order of ± 3–5%. Fig. 4a,b shows the time-resolved "fast", oscillating components of the experimentally recorded responses, as well as the simulation results. Good agreement between the simulation results and the experiment makes it possible to further use the obtained parameters in the development of optoacoustic and MEMS devices using graphitized layers in diamond.

The response spectra are shown in Fig. 5ab. A characteristic feature of the position of the spectral lines is that they cannot be compared with the reciprocal propagation time of elastic pulses in any of the layers or their combination. For each of the layers, one can set the problem of eigen oscillations, but the boundary conditions must take into account the presence of other layers in the structure. Such a problem for the case of two layers on a substrate was solved in [29]. Using the results of [29], eigen oscillations of the structure (diamond cap / graphitized layer / diamond bulk) were calculated. The corresponding frequencies are shown by vertical arrows in Fig. 5a,b.

The optical response of a layered structure can be approximately represented as the sum of the [28] contributions proportional to the movement of the sample surface, displacement of the layers' boundaries, and photoelastic interaction in layers and diamond bulk.

Simulation makes it possible to elucidate the nature of spectral lines. For example, in the region with a dose of 4·10^15^ cm^−2^ (Fig. 5b), the 28 and 51.5 GHz lines appear due to the movement of the diamond cap surface. A change in the thickness of the diamond cap layer also makes a contribution to the phase channel at a frequency of 28 GHz, but with the opposite sign, as a result, this line disappears. Some calculated eigen frequencies, for example, 71.4 GHz, practically do not appear in the experimental response spectrum, either in amplitude or in phase. This is due to the fact that the contributions from the surface motion and from the photoelastic interaction in the graphitized layer at this frequency have opposite signs. The 107.1 GHz line is caused by the photoelastic interaction in the diamond cap.

Similarly, in the region with a dose of 12·10^15^ cm^−2^, there is no 61 GHz line, and the 21 GHz and 45.5 GHz lines are present only in the phase channel.

The given examples illustrate the different "optical activity" of the vibrational modes of the structures under study. A similar effect was observed previously on GaAs membranes [30].

## 5 Conclusions

The acoustic properties of graphitized layers buried in diamond by ion implantation were studied using the picosecond ultrasonic technique. The wave resistance of such layers is approximately three times less than that of diamond. It has been established that with an increase in the implantation dose in the range from 4∙10^15^ cm^−2^ to 12∙10^15^ cm^−2^, the wave resistance of the layers decreases by 20%.

In addition, a comparison of experimental data with simulation results showed that, starting from a dose of 10∙10^15^ cm^−2^, the layers cannot be considered acoustically uniform in thickness, as the wave impedance changes by more than 20% within the layer, reaching a minimum approximately in the middle, in the region with maximum radiation damage.

The graphitized layer's longitudinal modulus of elasticity was determined depending on the dose of implantation. Our estimates show that the length of the viscoelastic damping in the graphite layer is 1.6∙10^18^/ω^2^ [m], which corresponds to the phonon lifetime at a frequency of 100 GHz, approximately 400 ps.

The generation of coherent phonons by graphitized layers under femtosecond laser irradiation has been studied. Phonons are generated with frequencies ranging from approximately 20 to 100 GHz, depending on the layer depth and thickness. It has been found that different vibration modes of diamond structures with graphitized layers have different "optical activity," which means that individual modes may not contribute to the optical response.

The studies performed have shown that even in cases when the length of elastic pulses is greater than the characteristic dimensions of structural inhomogeneities, it is possible to determine their parameters with high accuracy. By combining acoustic depth profiling and scanning along the structure's surface, one can realize hypersound tomography.

The large difference between the wave resistance of graphitized layers and diamond makes them promising in terms of potential applications in acoustoelectronic devices and microwave MEMS based on diamond. Our studies show that graphitized layers can also be used as an efficient generator of elastic pulses embedded into diamond with durations of up to a few picoseconds.

## Declaration of Competing Interest

The authors declare that they have no known competing financial interests or personal relationships that could have appeared to influence the work reported in this paper.

## Data Availability

Data will be made available on request.
